# Novel Immune-Related Genetic Expression for Primary Sjögren's Syndrome

**DOI:** 10.3389/fmed.2021.719958

**Published:** 2022-01-03

**Authors:** Jiajia Cui, Hui Li, Tianling Wang, Qin Shen, Yuanhao Yang, Xiujuan Yu, Huaixia Hu

**Affiliations:** Department of Rheumatology and Immunology, East Hospital of the Second People's Hospital of Lianyungang City, Lianyungang, China

**Keywords:** primary Sjögren's syndrome, weighted gene co-expression network analysis, immune cell, protein–protein interaction network, support vector machine model

## Abstract

**Objective:** To identify novel immune-related genes expressed in primary Sjögren's syndrome (pSS).

**Methods:** Gene expression profiles were obtained from the Gene Expression Omnibus (GEO) database, and differentially expressed genes (DEGs) were screened. The differences in immune cell proportion between normal and diseased tissues were compared, weighted gene co-expression network analysis was conducted to identify key modules, followed by a protein–protein interaction (PPI) network generation and enrichment analysis. The feature genes were screened and verified using the GEO datasets and quantitative real-time PCR (RT-qPCR).

**Results:** A total of 345 DEGs were identified, and the proportions of gamma delta T cells, memory B cells, regulatory T cells (Tregs), and activated dendritic cells differed significantly between the control and pSS groups. The turquoise module indicated the highest correlation with pSS, and 252 key genes were identified. The PPI network of key genes showed that *RPL9, RBX1*, and *RPL31* had a relatively higher degree. In addition, the key genes were mainly enriched in coronavirus disease-COVID-2019, hepatitis C, and influenza A. Fourteen feature genes were obtained using the support vector machine model, and two subtypes were identified. The genes in the two subtypes were mainly enriched in the JAK-STAT, p53, and toll-like receptor signaling pathways. The majority of the feature genes were upregulated in the pSS group, verified using the GEO datasets and RT-qPCR analysis.

**Conclusions:** Memory B cells, gamma delta T cells, Tregs, activated dendritic cells, *RPL9, RBX1, RPL31*, and the feature genes possible play vital roles in the development of pSS.

## Highlights

- The proportions of four immune cells were significantly different between the control and pSS groups.
- *RPL9, RBX1*, and *RPL31* had relatively higher degrees in the PPI network of key genes.
- Fourteen feature genes were obtained that were verified using GEO datasets and RT-qPCR.

## Introduction

Primary Sjögren's syndrome (pSS) is an autoimmune disease characterized by focal lymphocytic infiltration of the exocrine glands causing dry eyes and dry mouth (1). Although dryness is the main symptom, some pSS patients also experience systemic manifestations (2, 3). pSS can affect 0.1–0.6% of the general adult population, with a female-to-male predominance of 9:1, and the average age at diagnosis being approximately 50 years (4–6). The underlying causes and pathogenesis of pSS are not clear, making its effective treatment an enduring clinical challenge.

Several factors are expected to determine the occurrence of pSS: genetic predisposition, infection, and endocrine factors can work together to abnormally activate innate or adaptive immunity, resulting in the production of cytokines and antibodies, and lymphocyte infiltration (7, 8). Several studies have elucidated the genetics of pSS. Lin et al. found that the expression of several pSS-associated candidate genes, including *CXCL9, CXCL13*, and *PTPRC*, was upregulated (9). Inamo et al. identified differential expression levels of *SOX4* between patients with pSS and healthy controls (10). Zhang et al. indicated that the expression of some pSS-associated genes, for example *TAP2, IFI16*, and *HLA-DRA*, was upregulated (11). Vitali et al. found that *IFNG, TRIM26*, and *EDN1* were overexpressed in pSS patients (12). Although studies have linked pSS to the immune system response *in vivo* (13, 14), there is a lack of novel immune-related genes to study the underlying causes and inform treatment.

**Figure 1:**
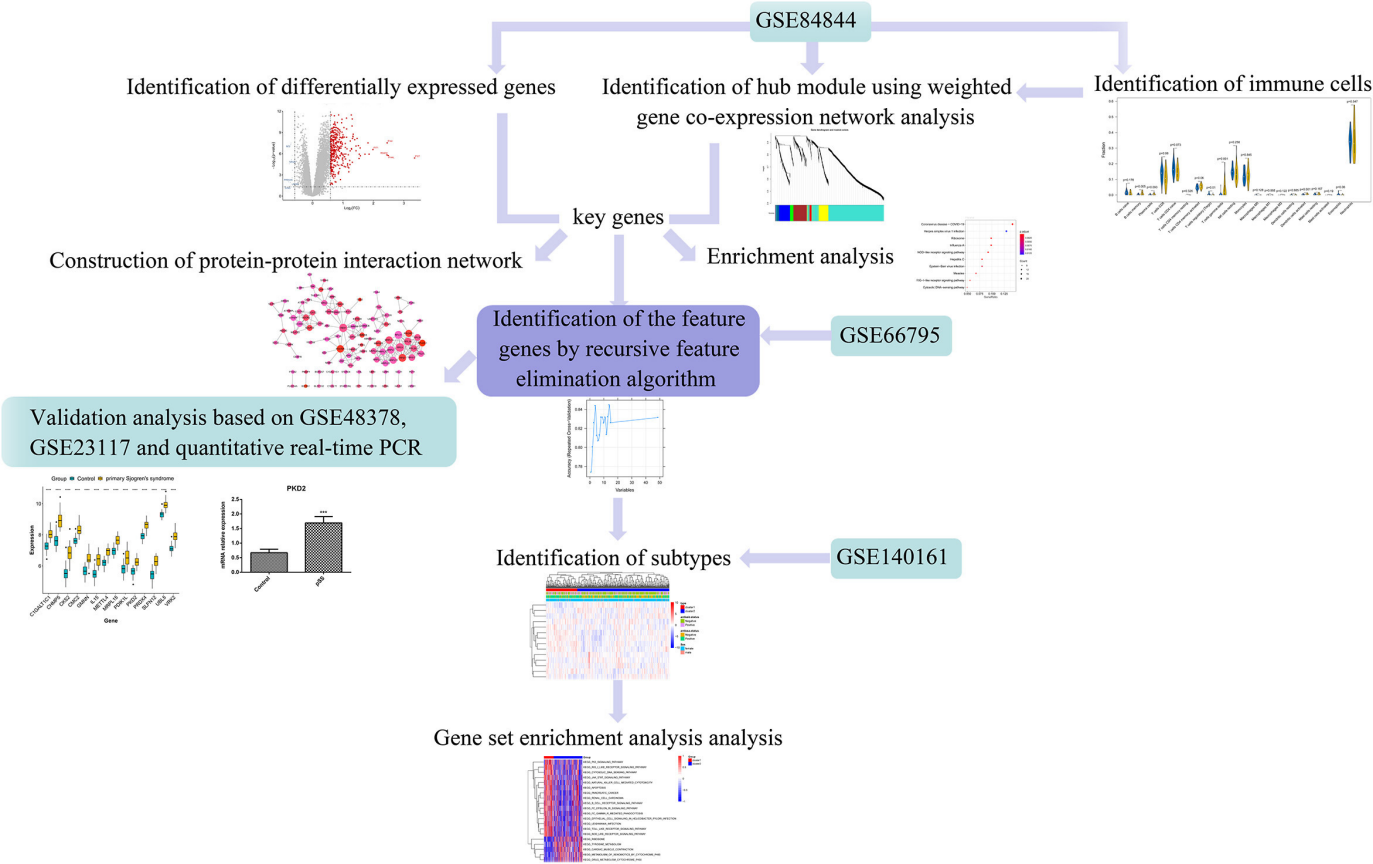
Workflow of this study. Differentially expressed genes (DEGs) were screened based on the gene expression profiles obtained from the Gene Expression Omnibus (GEO) database. The differences in respective immune cell proportions between control and diseased tissues were compared. Weighted gene co-expression network analysis (WGCNA) was performed to identify the key modules, followed by a protein-protein interaction (PPI) network generation and enrichment analysis. The feature genes were screened using the recursive feature elimination algorithm and verified using the GEO datasets and quantitative real-time PCR (RT-qPCR).

Using weighted gene co-expression network analysis (WGCNA), Yao et al. identified key genes and pathways in SS (15). Lei and Zhang identified key genes and pathways involved in B cells in patients with pSS (16). However, these studies used fewer datasets with a small sample-size and conducted no experimental validation. Therefore, in the present study, we aimed to identify novel immune-related genes in pSS using several bioinformatic approaches to ultimately inform the research and development of pSS therapies. The workflow of this study is illustrated in Figure 1. First, differentially expressed genes (DEGs) were screened based on the gene expression profiles obtained from the Gene Expression Omnibus (GEO) database. The differences in immune cell proportions were compared between normal and diseased tissues, and WGCNA was conducted to identify the key modules. The key genes were identified, followed by a protein–protein interaction (PPI) network generation and enrichment analysis. Finally, the feature genes were screened by a recursive feature elimination algorithm and verified using the GEO datasets and quantitative real-time PCR (RT-qPCR).

## Materials and Methods

### Microarray Data

The gene expression profiles of the datasets GSE84844, GSE66795, GSE140161, GSE48378, and GSE23117 were acquired from the GEO database. GSE84844 included data for whole blood samples from 30 patients with pSS and 30 healthy volunteers and was analyzed using the GPL570 [HG-U133_Plus_2] Affymetrix Human Genome U133 Plus 2.0 Array. GSE66795 contained data for 131 whole blood samples from patients with fully phenotyped pSS and 29 healthy controls and was analyzed using the GPL10558 Illumina HumanHT-12 v4.0 expression beadchip. GSE140161 contained data for 351 whole blood samples from 30 patients with pSS and was analyzed using the GPL23159 platform [Clariom_S_Human] Affymetrix Clariom S Assay, human (includes Pico Assay). GSE48378 contained data for peripheral blood mononuclear cells from 11 patients with pSS and 16 healthy controls and was analyzed using the GPL5175 [HuEx-1_0-st] Affymetrix Human Exon 1.0 ST Array [transcript (gene) version] platform. GSE23117 contained data for minor salivary gland samples from 10 patients with SS and 5 controls and was analyzed using the GPL570 [HG-U133_Plus_2] Affymetrix Human Genome U133 Plus 2.0 Array.

### Data Preprocessing and Identification of DEGs

The preprocessed and standardized probe expression value matrices of the four datasets were downloaded from the GEO database. The probes were annotated based on the annotation files. The probes that did not match any gene symbols were removed. When multiple probes matched one gene symbol, the mean value was selected as the expression value. Based on the GSE84844 dataset, the DEGs between the pSS patients and healthy controls were screened using the classical Bayesian method of the R “limma” package (17) (https://cran.r-project.org). The Benjamini–Hochberg method was used to correct the *P*-value. The threshold values of the DEGs were set as adjusted *P*-value < 0.05 and |logFC| > 0.585.

### Immune Infiltration Estimation

Based on the expression profile of the GSE84844 dataset, we used the CIBERSORT algorithm (18) with the parameters perm = 100 and QN = F to estimate the relative proportions of 22 immune cells. The proportions of the respective immune cells were then compared between the normal and diseased samples. The EPIC online tool (https://gfellerlab.shinyapps.io/EPIC_1-1/) was also utilized to evaluate the proportions of immune cells.

### WGCNA

We identified the modules related to pSS using the WGCNA package (19) in R. The procedures for network construction and module identification consisted of estimating the gene co-expression correlation matrix, defining the adjacent function, determining the phase difference between nodes, and identifying correlation among network module, disease, and relative abundance of different immune cells. Then, the phenotype–immune cell related modules were obtained.

### Construction of the PPI Network

The key genes were identified as those overlapping when intersecting the DEGs with the genes in the phenotype–immune cell-related modules. The PPIs of the key genes were screened using the STRING database (20), with the species as “human” and a PPI score of 0.9. Using Cytoscape (21), the PPI network was constructed, and the nodes with high degrees were considered as hub genes. Moreover, to elucidate the functional role of the key genes in pSS, enrichment analysis was performed using a clusterProfiler tool (22) with a threshold *P*-value < 0.05 and number of enriched genes ≥2.

### Identification of Feature Genes

A random forest model was established based on the key genes, and the top 50 genes were obtained. Based on the GSE66795 dataset, the recursive feature elimination algorithm of the caret package (23) in R and the repeated CV sampling method were used to identify the feature genes. Based on the feature genes, a support vector machine (SVM) (23) model was constructed using the GSE66795 dataset with the default parameters. A receiver operating characteristic (ROC) curve was used to evaluate the accuracy of the model by the area under the curve (AUC) estimate.

### Identification of Subtypes Based on the GSE140161 Dataset

Based on the centered Pearson correlation algorithm, the pheatmap package in R was used to perform bidirectional hierarchical clustering, and an unsupervised cluster analysis was conducted on the obtained feature genes based on the GSE140161 dataset to identify the subtypes. A heatmap of the clinical characteristics of the subtypes was computed. The gene set file c2.cp.kegg.v7.2.symbols.gmt from the Molecular Signatures Database v7.2 was used as an enrichment background, the GSVA algorithm (24) in R was employed to perform the enrichment analysis, with the threshold set according to the adjusted *P*-value < 0.05, and the Benjamini-Hochberg method was used to correct the *P*-value.

### Validation Analysis

To verify the identified feature genes, their expression levels in the GSE48378, GSE23117, and GSE140161 datasets were used as feature values, and the SVM model was constructed with the default parameters. To evaluate the accuracy of the model, the ROC curve was constructed. The expression level of the 14 feature genes was evaluated in the GSE48378 dataset. Additionally, we performed RT-qPCR to verify the expression levels of these feature genes. A total of 20 whole blood samples from 10 pSS patients and 10 healthy controls were obtained from the Lianyungang Second People's Hospital. The study group was determined by the following criteria: (a) patients who met the diagnostic criteria for the 2002 international classification of SS were included; (b) patients receiving moderate to high doses of corticosteroids, immunosuppressants, or biologics were excluded. The control group consisted of healthy individuals showing no symptoms for SS or other autoimmune-related diseases, with no family history of the disease, and were not currently receiving any medication. This study was approved by the ethics committee of the Lianyungang Second People's Hospital (approval number: 2021K005). Written consent was provided by all of the participants in this study. RT-qPCR was performed as described previously (25). All experiments were performed in triplicate. *GAPDH* was used as an internal control. A *t*-test was used for comparisons of means between the groups. The primers used in this study are listed in Table 1.

**Table 1:** Primers used in this study.

**Gene name**	**Forward primer (5^′^-3^′^)**	**Reverse primer (5^′^-3^′^)**
*CHMP5*	AGATTTCTCGATTGGATGCTGAG	TGTTGGGCAAGATTGTCCCG
*SLFN12*	TTGGAAACGAATTATGCCGAGT	AGAGCACACATAGCTCGTGAG
*IL15*	CATCCATCTCGTGCTACTTGTG	GCCTCTGTTTTAGGGAGACCT
*VRK2*	GTGGATAGAACGCAAACAACTTG	CGGATACCTAATTGCAGGACAGT
*GMNN*	GCCCTGGGGTTATTGTCCC	AGCGCCTTTCTCCGTTTTTCT
*CKS2*	TTCGACGAACACTACGAGTACC	GGACACCAAGTCTCCTCCAC
*PRDX4*	AGAGGAGTGCCACTTCTACG	GGAAATCTTCGCTTTGCTTAGGT
*UBL5*	GGGAAGAAGGTCCGCGTTAAA	ACGTGGTCCTTAAAAATCGTGT
*CMC2*	CCTGACTTATCTCCACACTTGC	TCTCAACTCCCGATCAACATCA
*MRPL15*	GGCTCCAAGAAACCGGAGAG	GCGTCTGAAACTATGTCCTTCGT
*PKD2*	CTCTGGGGAACAAGACTCATGG	TCATCATGCCGTAGGTCAAGA
*PDIK1L*	ATGGTGAGTAGCCAGCCAAAG	CTGCTTCATACACAACACCGTA
*METTL4*	TCTGTGGTACACCAGTTGTCA	CCTTTTTACGGCAACAAGGTTCA
*C1GALT1C1*	AGTTTGCCTGAAATATGCTGGA	GGGGTGATAAGTCATTGCCTCT
*GAPDH*	CAGCCTCAAGATCATCAGCA	GGATCTCGCTCCTGGAAGATG

## Results

### Identification of DEGs and Immune Infiltration Estimation

**Figure 2:**
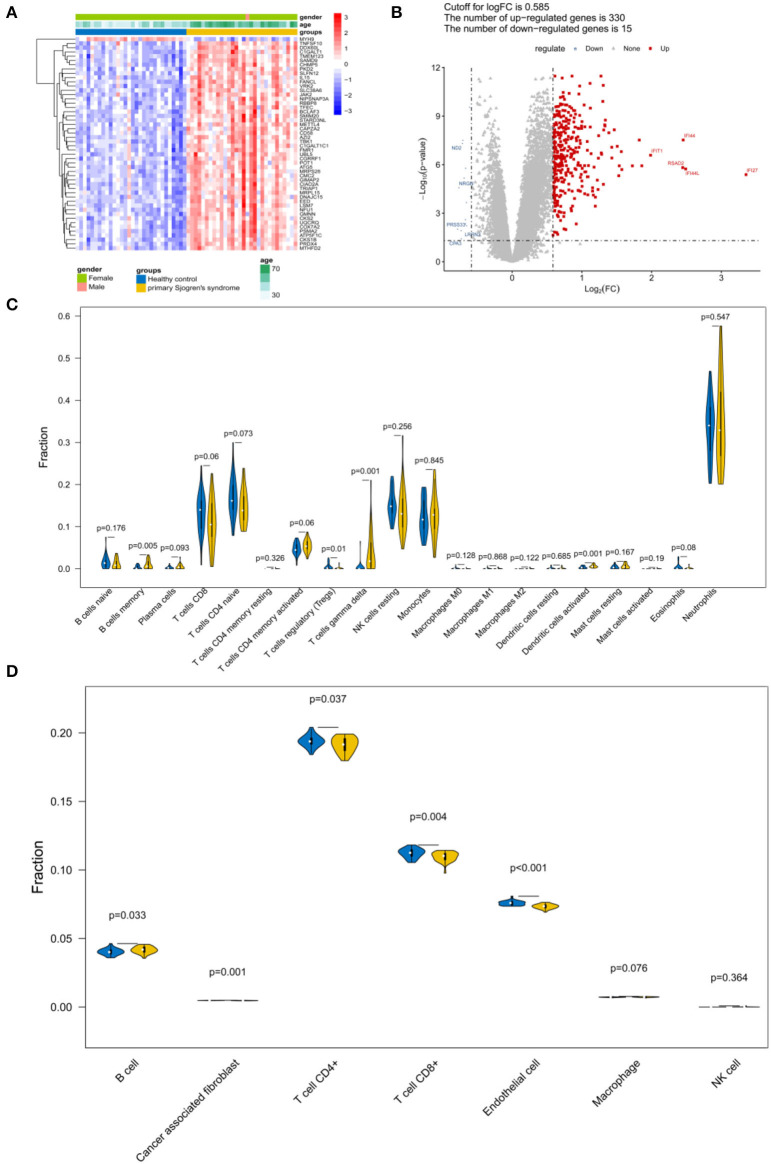
Identification of differentially expressed genes (DEGs) and immune infiltration estimation. **(A)** Heatmap and **(B)** volcano plot of the DEGs. **(C)** Violin plot depicting the abundance distribution of 20 immune infiltrating cell types identified using the CIBERSORT algorithm. **(D)** Violin plot of the abundance distribution of seven types of immune cells identified using the EPIC tool. The blue strip represents the normal control group and the yellow strip represents the primary Sjögren's syndrome (pSS) group.

A total of 345 DEGs were obtained between the samples from patients with pSS and the healthy controls (Figures 2A,B; Supplementary File 1). Because follicular helper T cells and activated NK cells were not observed, the relative abundance of 20 immune cells was obtained for each sample. According to the CIBERSORT algorithm, the abundance distribution of memory B cells, regulatory T cells (Tregs), gamma delta T cells, and activated dendritic cells differed significantly between the control and pSS groups (*P* < 0.05; Figure 2C). According to the EPIC tool analysis results (Figure 2D), the abundance distributions for B cells, cancer associated fibroblasts, T cells, and endothelial cells differed significantly between the control and pSS groups (*P* < 0.05).

### WGCNA and Module Identification of pSS

**Figure 3:**
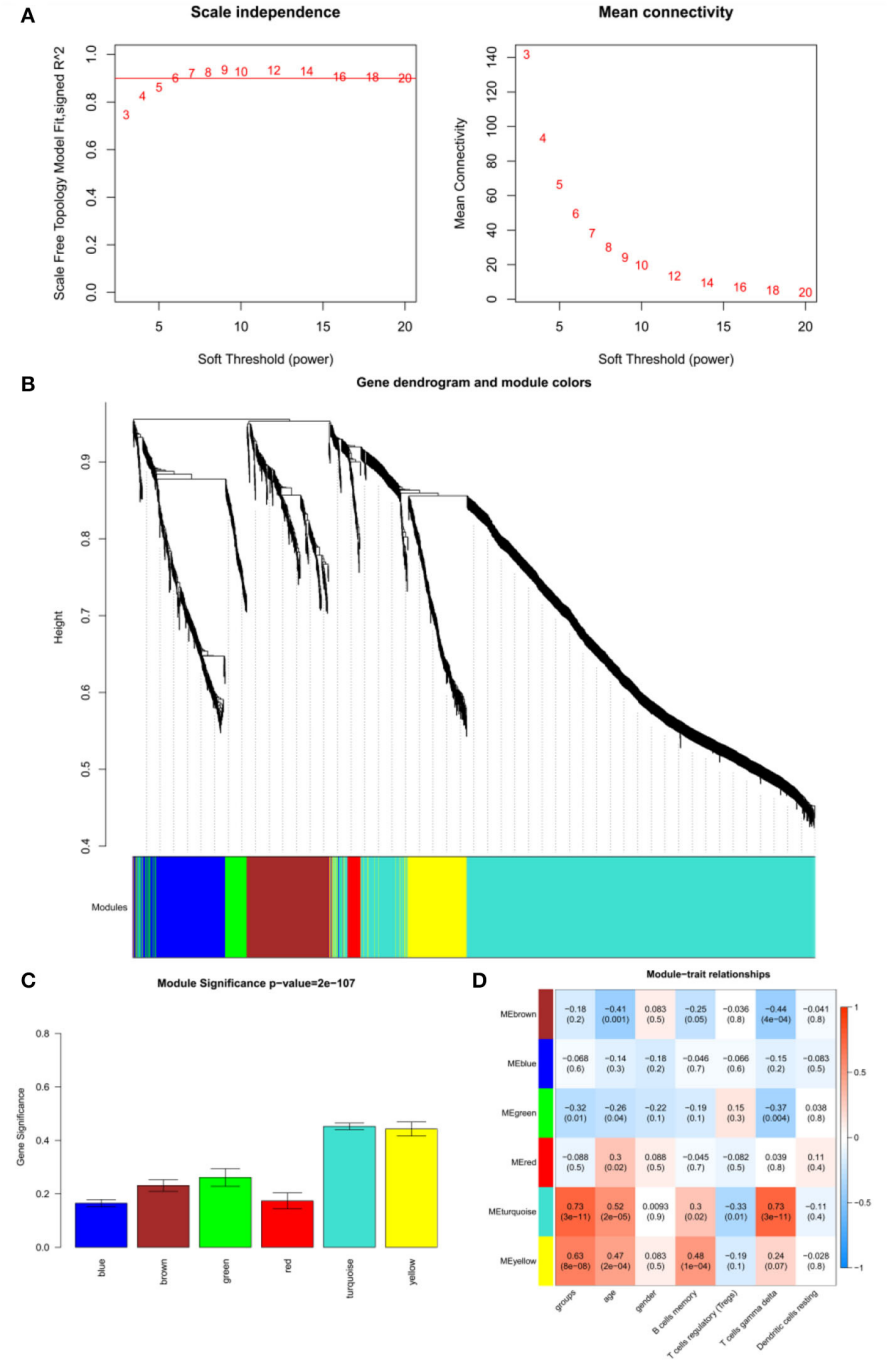
Results of weighted gene co-expression network analysis (WGCNA). **(A)** Determination of the soft threshold for adjacency matrix. The horizontal axis represents the soft threshold power, and the vertical axis represents the square of the correlation coefficient of log_2_k and log_2_p(k). The red line indicates the location when the correlation coefficient is 0.9, and the corresponding soft threshold power is 6. **(B)** Gene dendrogram derived from hierarchical clustering. The different modules are indicated by colors underneath the dendrogram. **(C)** Correlations between modules and diseases. The horizontal axis represents modules, and the vertical axis represents the overall correlation coefficient between genes and disease states in the module. **(D)** Correlations between modules and traits. The upper figure in each row represents the correlation whereas the lower figure represents the *P*-value. Blue represents a negative correlation, and red represents a positive correlation.

The adjacency matrix weighting parameter power was analyzed to meet the prerequisite of a scale-free network distribution. The power of β = 6, where the square of the correlation coefficient between log2k and log2p(k) = 0.9, was selected as the soft thresholding parameter (Figure 3A). To obtain a system clustering tree of the DEGs, combined with the dissimilarity matrix, hierarchical clustering was conducted. A total of six modules were screened, with the least number of genes set as 30 and the pruning height set as cutHeight = 0.3 (Figure 3B). Among the six modules, the turquoise and yellow modules had the highest association with pSS (Figure 3C). The gene significance was calculated to identify the modules that were highly correlated with pSS, and the results showed that the turquoise module had the highest correlation with pSS (Figure 3D). Thus, the turquoise module was selected for further analysis.

### Construction of the PPI Network

**Figure 4:**
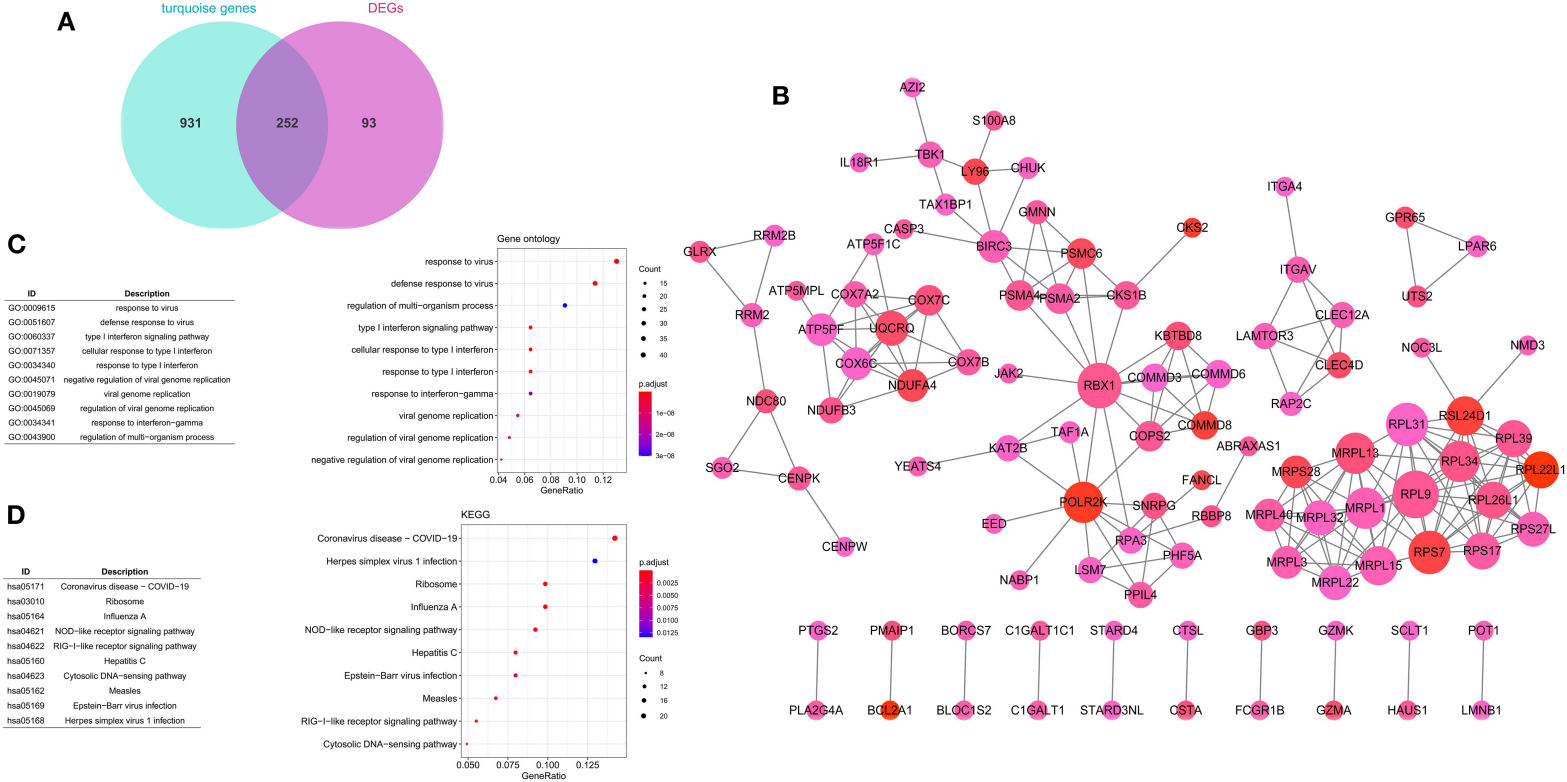
Construction of protein–protein interaction (PPI) network. **(A)** Venn diagram of key genes. **(B)** PPI network of key genes. The large nodes have a higher degree, and red nodes have a large log FC. The Gene Ontology (GO) **(C)** and Kyoto Encyclopedia of Genes and Genomes (KEGG) **(D)** pathway enrichment analyses of key genes. Circle colors represent *P*-values as indicated. The circle size represents the GeneRatio, which is the ratio of the number of genes enriched in a certain pathway to the number of genes in the group.

A total of 252 key genes were screened (Figure 4A; Supplementary File 2); the PPI network of the key genes (Figure 4B) included 102 nodes and 210 interactions. *RPL9, RBX1*, and *RPL31* showed a relatively higher degree in the PPI network (Table 2), and were identified as hub genes. The enrichment analysis showed that the key genes were involved in 104 Gene Ontology (GO)-biological processes (BP) (Figure 4C), such as cellular response to interleukin-1 (involving *RBX1*) and response to interleukin-1 (involving *RBX1*), and 23 Kyoto Encyclopedia of Genes and Genomes (KEGG) pathways, including coronavirus disease (COVID-19) (involving *RPL9* and *RPL31*), hepatitis C, influenza A, and ribosome (involving *RPL9* and *RPL31*) (Figure 4D; Supplementary Files 3, 4).

**Table 2:** Degree of proteins (top 10) in the protein–protein interaction (PPI) network.

**Proteins**	**Betweenness**	**Closeness**	**Degree**	**logFC**
RPL9	68.27821	0.012041	14	0.837228
RBX1	733.3333	0.014844	13	0.857245
RPL31	25.56026	0.012038	12	0.614054
RPS7	25.56026	0.012038	12	1.174916
RPL34	16.13462	0.012037	11	0.894338
MRPL13	26.70256	0.012034	11	0.959121
MRPL1	26.70256	0.012034	11	0.69091
POLR2K	412.3333	0.014816	11	1.336471
RSL24D1	70	0.012031	10	1.236482
MRPL15	18.27692	0.012032	10	0.692194

### Identification of the Feature Genes

**Figure 5:**
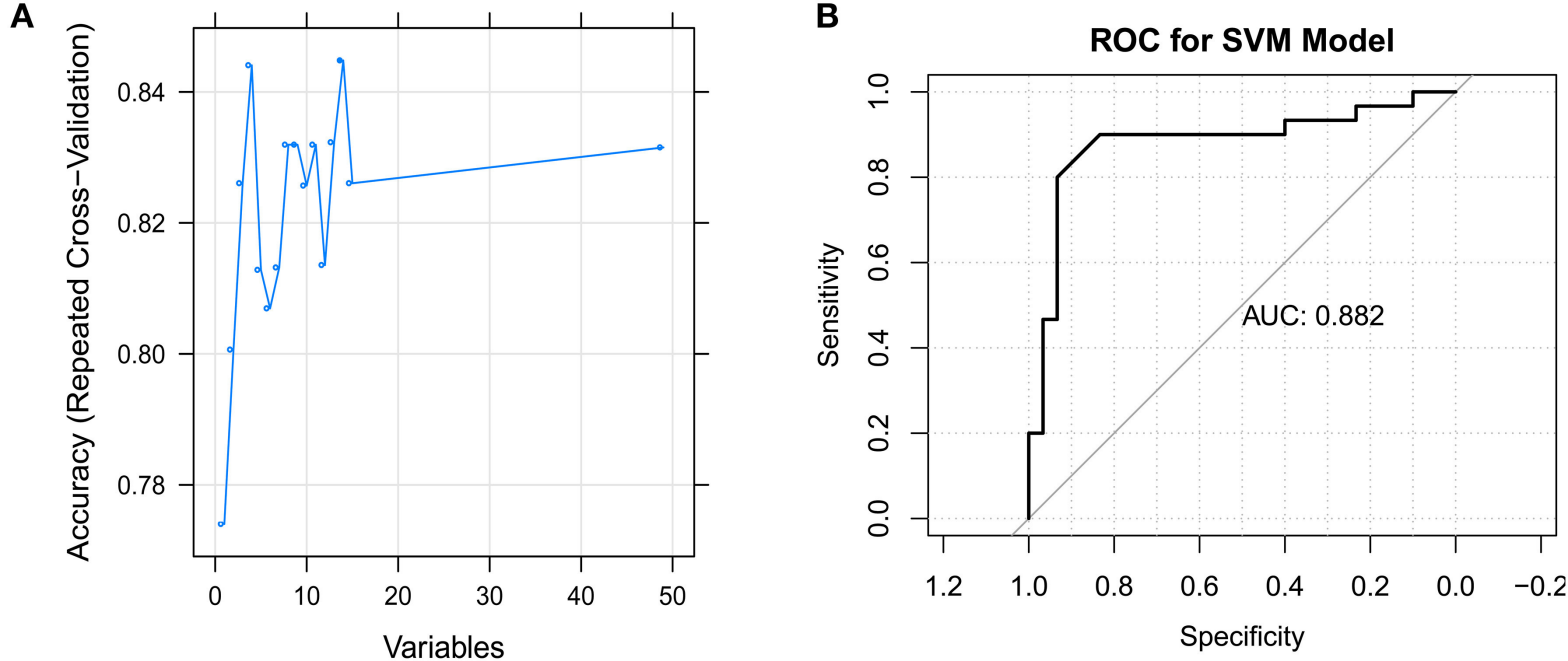
Identification of the feature genes. **(A)** The support vector machine (SVM) mode screens 14 feature genes using the RFE algorithm. **(B)** Receiver operating characteristic (ROC) curve for the SVM model.

The SVM model was constructed using the samples in the GSE66795 dataset with default parameters based on the top 50 genes obtained using the recursive feature elimination algorithm. There were 14 feature genes in the SVM model: *CHMP5, SLFN12, IL15, VRK2, GMNN, CKS2, PRDX4, UBL5, CMC2, MRPL15, PKD2, PDIK1L, METTL4*, and *C1GALT1C1* (Figure 5A). The AUC was 0.882 (Figure 5B).

### Identification of Subtypes Based on the GSE140161 Dataset

**Figure 6:**
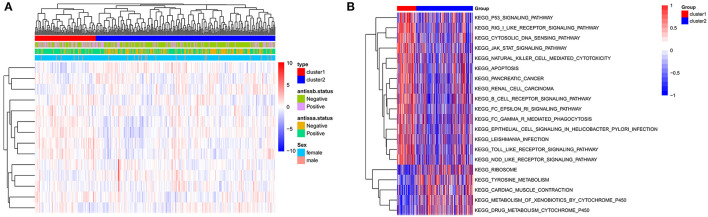
Identification of subtypes based on the GSE140161 dataset. **(A)** Heatmap of subtypes and clinical information. **(B)** Gene set enrichment analysis of genes in the two subtypes.

According to the unsupervised cluster analysis, two subtypes were screened: Cluster 1 and Cluster 2. Bidirectional hierarchical clustering was performed to generate a heatmap of the clinical characteristics of the subtypes (Figure 6A). Gene set enrichment analysis (GSEA) showed that the genes related to the subtypes were enriched in 50 KEGG pathways, including the p53, JAK-STAT, and toll-like receptor signaling pathways (Figure 6B).

### Validation Analysis

**Figure 7:**
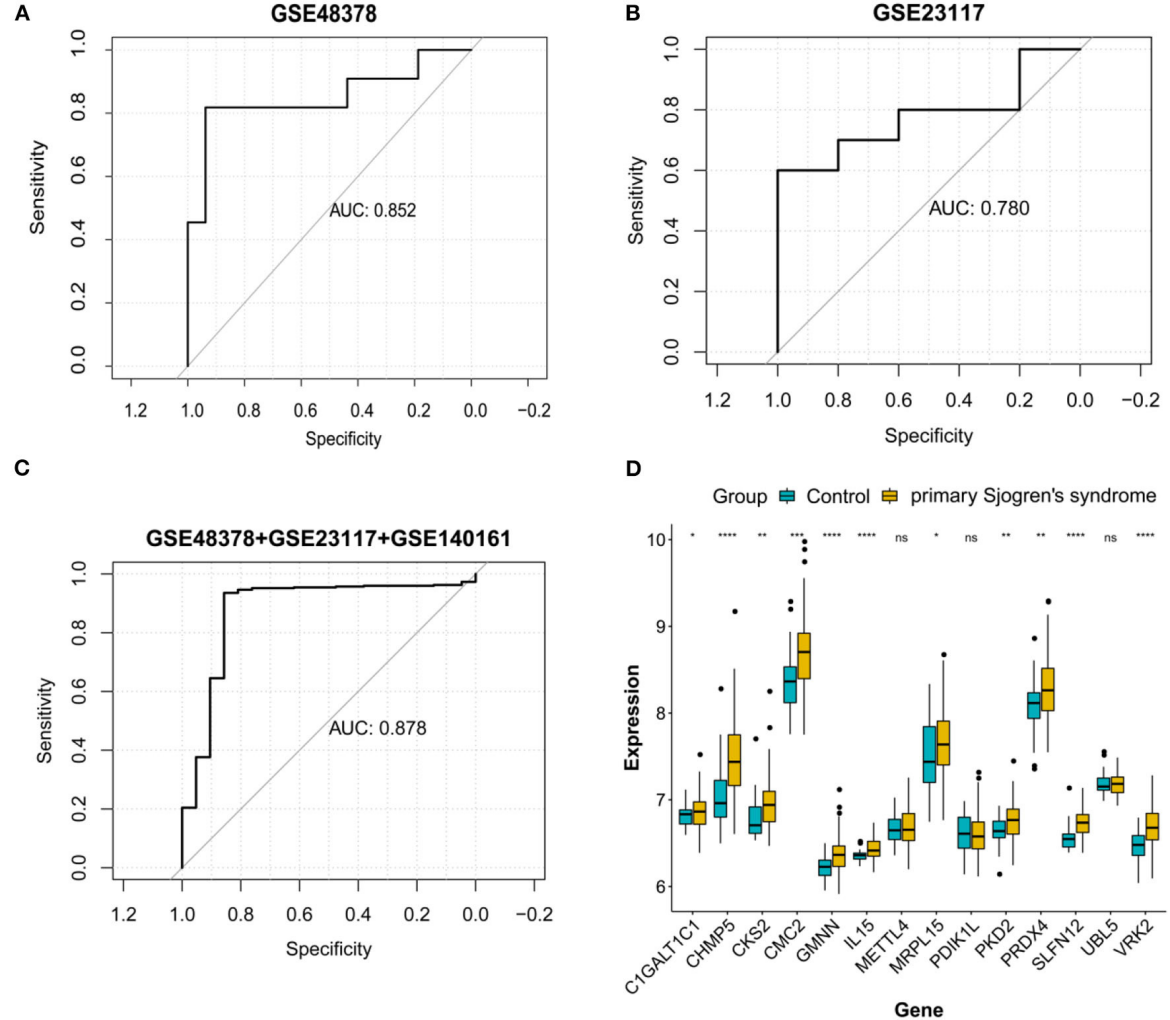
Validation analysis in the Gene Expression Omnibus (GEO) datasets. Receiver operating characteristic curve for the feature genes in the GSE48378 dataset **(A)**, GSE23117 dataset **(B)**, and GSE48378 + GSE23117 + GSE140161 datasets **(C)**. **(D)** The expression level of the 14 feature genes in the GSE48378 dataset. The blue strip represents the normal control group and the yellow strip represents the primary Sjögren's syndrome (pSS) group. *P < 0.05; **P < 0.01; ***P < 0.001; ns indicates with no statistical significance.

**Figure 8:**
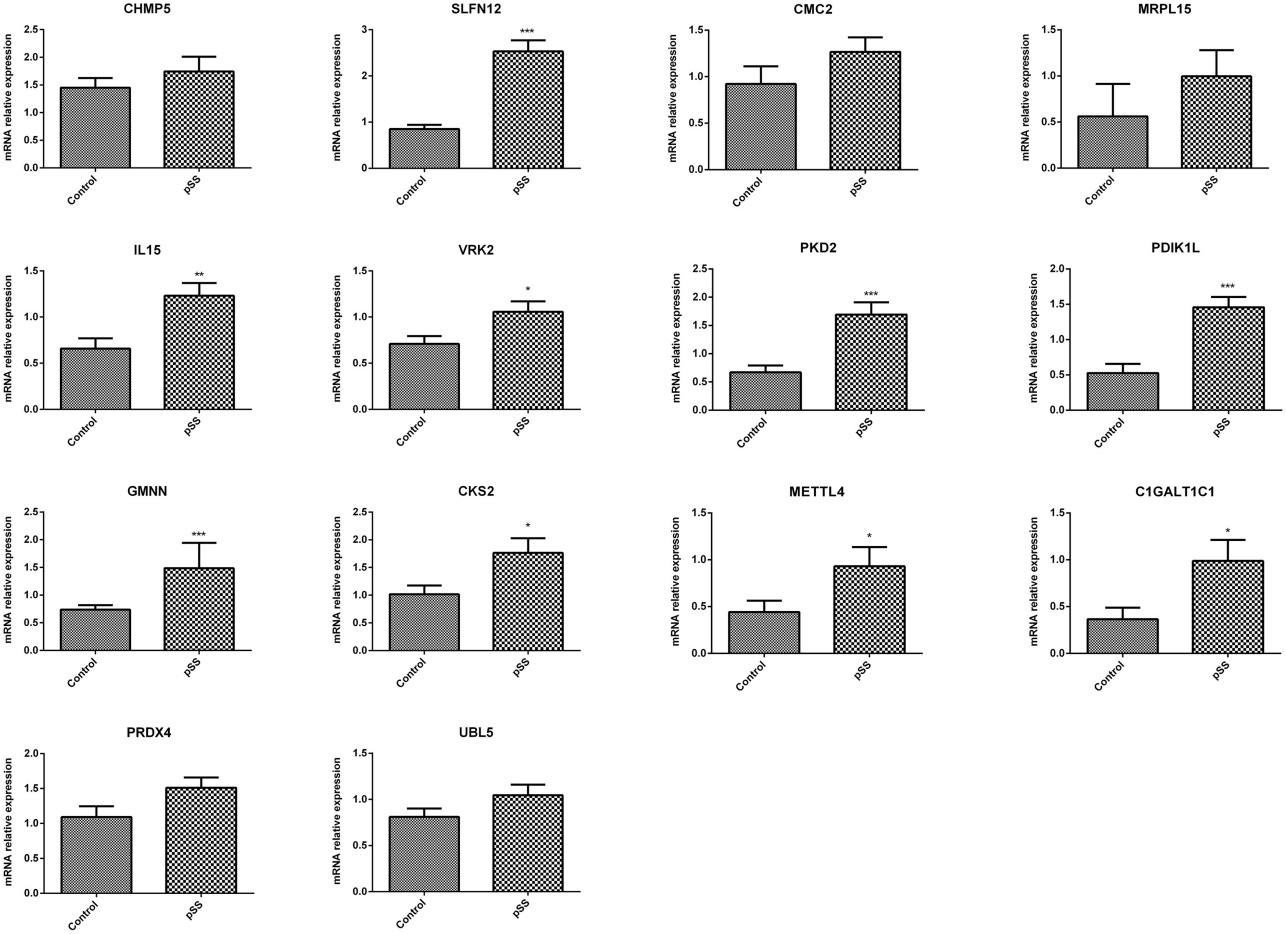
Quantitative real-time PCR (RT-qPCR) analysis to verify the feature genes. *P < 0.05; **P < 0.01; ***P < 0.001.

ROC curve analyses estimated an AUC of 0.852 in the GSE48378 dataset (Figure 7A), 0.780 in the GSE23117 dataset (Figure 7B), and 0.878 in the GSE48378 + GSE23117 + GSE140161 datasets (Figure 7C). The majority of the 14 feature genes were upregulated in the pSS group of the GSE48378 dataset (Figure 7D). According to RT-qPCR analyses, all the genes were upregulated in the pSS group, and significantly higher expression levels were observed for *SLFN12, IL15, VRK2, GMNN, CKS2, PKD2, PDIK1L, METTL4*, and *C1GALT1C1* in the pSS group compared to the control group (*P* < 0.05, Figure 8).

## Discussion

SS is an autoimmune exocrine disease characterized by chronic inflammation and destruction of the salivary and lacrimal glands (3). Therefore, based on a large-scale SS cohort, combined with the identification of immune-related genes, effective immune markers were screened in this study. A total of 345 DEGs were identified, and the proportions of memory B cells, Tregs, gamma delta T cells, and activated dendritic cells differed significantly between the control and pSS samples. The turquoise module had the highest correlation with pSS, and 252 key genes were identified. The PPI network of the key genes showed that *RPL9, RBX1*, and *RPL31* had a relatively higher degree. In addition, the key genes were involved in 104 GO-BPs and 23 KEGG pathways, including COVID-19, hepatitis C, influenza A, and ribosome. A total of 14 feature genes were identified in the SVM model: *CHMP5, SLFN12, IL15, VRK2, GMNN, CKS2, PRDX4, UBL5, CMC2, MRPL15, PKD2, PDIK1L, METTL4*, and *C1GALT1C1*. Two subtypes were screened, and the feature genes in the two subtypes were enriched in 50 KEGG pathways. The 14 feature genes were finally verified using GEO datasets and RT-qPCR.

The importance of immune cells in the pathogenesis of pSS has been studied extensively. Hansen et al. reported that B cells are an indication of selection disorder and differentiation in ectopic lymphoid tissue in SS (26). The infiltration of B and T cells in the salivary gland and the formation of germinal center-like structures are characteristic of pSS (27). Dendritic cells are key candidates for activating T cells and B cells in pSS (28). Moreover, the abnormality of memory B cells seems to be closely related to the pathogenesis of pSS and its malignant complication, B-cell lymphoma (29). Although Tregs prevent autoimmunity and maintain immunological homeostasis, Bernard et al. found that Tregs were related to systemic autoimmune diseases, including pSS (30). Lamour et al. found that the proportions of both CD16^+^ and HLA-DR^+^ gamma delta T cells were significantly higher in patients with pSS than in controls (31). In this study, the proportions of 20 immune cells were evaluated using the CIBERSORT algorithm, Among them, the proportions of gamma delta T cells, memory B cells, Tregs, and activated dendritic cells differed significantly between the control and pSS groups. According to analyses with the EPIC tool, the proportions of B cells, cancer associated fibroblasts, T cells, and endothelial cells differed significantly between the control and pSS groups. Both analyses indicated significant differences between the control and pSS groups, with respect to immune cell composition. Taken together, our results suggest that these immune cells play vital roles in the pathogenesis of pSS.

A total of 252 key genes were identified in this study, and *RPL9, RBX1*, and *RPL31* were identified as hub genes according to the PPI network. The key genes were involved in 23 KEGG pathways, including COVID-19 (involving *RPL9* and *RPL31*), hepatitis C, influenza A, and ribosome (involving *RPL9* and *RPL31*). Bosaeed and Kumar revealed that, compared with the general population, the risk of influenza infection in patients with autoimmune diseases, such as pSS, is significantly increased (32). Ferri et al. reported that patients with systemic autoimmune diseases were more susceptible to COVID-19 owing to a weakened immune system (33). Ramos-Casals et al. suggested that hepatitis C virus infections are related to the development of SS in specific subgroups of patients (34). Multiple defects in the process of ribosome production have been reported to cause a spectrum of human diseases (35). The strong association of pSS with infectious diseases has led to an enthusiastic scientific debate regarding the cause and pathogenesis, though understanding the underlying mechanisms will require innovative and intensive research. Although our study provides a promising baseline evaluation of pSS associated genes, the results need to be verified using appropriate wet-lab experiments. The GO functional analysis revealed that the key genes were also enriched in the cellular response to interleukin-1 (involving *RBX1*) and response to interleukin-1 (involving *RBX1*) terms. Yamada et al. revealed that interleukin-1 plays vital roles in the onset and development of SS by controlling systemic or local immune responses and maintaining the survival and mucosal defense of target epithelial cells (36). To date, there are no reports on *RPL9, RBX1*, and *RPL31* in pSS, and the potential correlations should be further evaluated.

In this study, 14 feature genes were identified—*CHMP5, SLFN12, IL15, VRK2, GMNN, CKS2, PRDX4, UBL5, CMC2, MRPL15, PKD2, PDIK1L, METTL4*, and *C1GALT1C1*—which were verified using several GEO datasets and RT-qPCR. The RT-qPCR results showed that the expression of all these genes was upregulated in the pSS group, and significantly higher expression levels of *SLFN12, IL15, VRK2, GMNN, CKS2, PKD2, PDIK1L, METTL4*, and *C1GALT1C1* were observed in the pSS group compared with the control group. *IL15*, also known as *IL-15*, is a multifunctional molecule with therapeutic potential and is a member of the immune regulatory cytokine family (37). Sisto et al. reported that *IL-15* is overexpressed at both mRNA and protein levels in pSS patients (38), which is consistent with the results of the present study. However, few studies have reported on the other feature genes identified in this study. According to the GSEA results, the feature genes in the two subtypes were enriched in 50 KEGG pathways, including the p53, JAK-STAT, and toll-like receptor signaling pathways. p53 is important in DNA repair, cell cycle arrest, hypoxia, and inflammation in various cells and tissues (39). Takatori et al. reported that p53 is supposedly involved in the pathogenesis of systemic autoimmune diseases in humans and mice (40). Aota et al. suggested that by regulating JAK/STAT signaling, baricitinib inhibited IFN-γ-induced CXCL10 expression and weakened immune cell chemotaxis, indicating its potential as a therapeutic strategy for pSS (41). Shimizu et al. indicated that toll-like receptor 7-dominant innate immunity is related to the development of sialadenitis in pSS (42). The present study has identified 14 feature genes that could play vital roles in the development of pSS via the p53, JAK-STAT, and toll-like receptor signaling pathways.

However, this study has some limitations. First, *RPL9, RBX1*, and *RPL31*, identified as hub genes in the PPI network, should be further verified in different datasets and using other relevant experiments. Second, as the immune cell composition of the samples was estimated using CIBERSORT algorithm and EPIC tool, further relevant experiments are required to verify their accuracy. Further experiments, using larger datasets, are needed to provide in-depth evaluations of the etiopathogenic basis of these genes in pSS.

## Conclusions

Several cells and genes (memory B cells, Tregs, gamma delta T cells, activated dendritic cells, *RPL9, RBX1, RPL31*, and feature genes) could play potential roles in the development of pSS. These findings add to our understanding of the pathogenesis of pSS and may inspire the development of new therapeutic approaches.

## Data Availability Statement

The original contributions presented in the study are included in the article/Supplementary Material, further inquiries that support the findings of this study are available in GEO database at (https://www.ncbi.nlm.nih.gov/geo/).

## Author Contributions

JC and HH conceptualized and designed the research and conceived of the study, participated in its design and coordination, and contributed to the writing and revision of the manuscript for important intellectual content. HL and XY contributed to data acquisition. TW participated in data analysis and interpretation. QS and YY designed the study and performed the statistical analysis. All authors read and approved the final manuscript.

## Conflict of Interest

The authors declare that the research was conducted in the absence of any commercial or financial relationships that could be construed as a potential conflict of interest.

## Publisher's Note

All claims expressed in this article are solely those of the authors and do not necessarily represent those of their affiliated organizations, or those of the publisher, the editors and the reviewers. Any product that may be evaluated in this article, or claim that may be made by its manufacturer, is not guaranteed or endorsed by the publisher.

## Abbreviations

AUC: Area under the curve
BP: Biological processes
GEO: Gene Expression Omnibus
GO: Gene Ontology
GSEA: Gene set enrichment analysis
KEGG: Kyoto Encyclopedia of Genes and Genomes
ROC: Receiver operating characteristic
SVM: Support vector machine
WGCNA: Weighted gene co-expression network analysis.
